# The shaping of the coherence function of resonate-and-fire neuron models

**DOI:** 10.1186/1471-2202-14-S1-P346

**Published:** 2013-07-08

**Authors:** Sven Blankenburg, Benjamin Lindner, Susanne Schreiber

**Affiliations:** 1 Bernstein Center for Computational Neuroscience, Berlin, 10115, Germany; 2 Institute for Theoretical Biology, Humboldt-Universität zu Berlin, Berlin, 10115, Germany; 3 Institute for Physics, Humboldt-Universität zu Berlin, Berlin, 12489, Germany

## 

It is known that integrate-and-fire neurons act as low-pass filters on information [1], i.e. they preferentially encode information at low frequencies. However, many neurons cannot be described by such integrators, even qualitatively. Experiments show that in several areas of the mammalian brain single neurons display a resonance property in their subthreshold voltage dynamics, see for example [2,3]. Here, we study the information transmission of a current-based resonate-and-fire neuron model [4] by means of the spectral coherence function. We use a stochastic input current (the Ornstein-Uhlenbeck process from statistical physics) to model a complex dynamical stimulus. We show by numerical simulations that resonate-and-fire neurons encode time-dependent stimuli preferentially at moderate frequencies, including their resonance frequency, i.e. the coherence function of this model shows a clear maximum as a function of frequency. This is in marked contrast to the low-pass coherence that is found for the pure subthreshold dynamics (in the absence of spiking) in spite of resonant filter properties. We discuss dynamical mechanisms that lead to the band-pass filtering of information in the spiking resonate-and-fire model.
